# Considerations in designing trauma-focused interventions for displaced Afghan women

**DOI:** 10.3389/fgwh.2022.893957

**Published:** 2023-02-23

**Authors:** Amaya Alexandra Ramos

**Affiliations:** Independent Scholar, Arlington, VA, United States

**Keywords:** Afghan women, refugee women, mental health, family systems, trauma-focused intervention, medical ethnography, post-traumatic stress disorder, participatory research

## Abstract

In light of the 2021 United States military withdrawal from Afghanistan, as well as the humanitarian crises of mass displacement and subsequent health system strain that have ensued, practitioners worldwide will need to develop a more nuanced understanding of the adverse life experiences that women from Afghanistan frequently endorse. As they bear a disproportionate impact of constraints within Afghan society, and as patriarchal systems affect most of their life domains, women from Afghanistan may present with high levels of baseline trauma upon resettlement, and health systems may seek to attenuate this distress; However, the nature of these traumatogenic events may shape women's receptivity to psychosocial interventions, particularly those which are at least partially rooted in Western modalities. In the absence of sufficient literature on evidence-based interventions for this population, a diversity of ethnographic and clinical literature is synthesized, including literature on interventions alleged to be compatible with Afghan norms. As it will be essential to support Afghan women's mental health following social reorganization on a massive scale, considerations arising from the interdisciplinary literature are offered so that they may inform the development of structured, trauma-focused interventions and so that the health systems with which they interface may be better prepared to serve them.

## 1. Introduction

Afghanistan has long been one of the largest originators of displaced persons due to prolonged interethnic conflict and proxy wars (1, 2); however, the year 2021 witnessed the U.S. military withdrawal from Afghanistan, the resultant collapse of the Afghan national infrastructure, and the subsequent takeover of the country by Taliban, Islamic State-Khorasan (ISK), and other terrorist forces (3), creating a renewed crisis of mass displacement.

The Soviet invasion of Afghanistan in 1979 incentivized a parallel escalation of indirect U.S. involvement, resulting in a protracted proxy-war that lasted until the time of the Soviet Union's withdrawal from Afghanistan in 1989 (2). The eventual waning of Soviet and U.S. influence allowed for the entrenchment of spiraling political conflict throughout the ensuing Afghan Civil War, one the outgrowths of which was the rise of the Taliban, providing part of the impetus for the U.S. invasion of Afghanistan in 2001. Yet, in addition to the U.S,' primary aims of countering Al-Qa'ida and the Taliban for the sake of global security (4), the occupation is stated to have had connections to multifaceted international tensions (2, 5), also yielding global economic incentives and rhetorical fodder around the rights of Afghan women (4, 6, 7). Prolonged occupation soon led to the country's near complete dependence on foreign systems due to a vast and complex ecosystem of global non-governmental actors, NATO-member militaries, and associated enterprises (4, 7–9) with sprawling “sub-contractual layers” (7).

When the U.S. military abruptly withdrew on 1 May 2021, the result was a gaping power vacuum that was rapidly filled by the Taliban and other non-state actors who overran Kabul by 15 August (10); however, the insurgents quickly came under severe international sanctions (11). Though the combination of the ensuing negotiations, administrative incompetence (12), and the looming threat of state failure modulated the insurgents' plans for total control (13), hostile forces continued to dismantle much of the progress attained during the previous two decades of reconstruction (12). Among other crimes, the Taliban continued to execute prominent public figures and introduced even greater restrictions on women's participation in society (14–16). Eventually, fears of reprisal as well as the total subjugation of women caused many Afghans flee by whatever means were available to them. This included seeking refuge in the NATO countries to which their families had connections. The result was that between 1 May and 30 August, 122,00 individuals of various nationalities had been airlifted out of Afghanistan (17), with others having sought refuge in third countries. The ensuing mass migration event has since placed great strain on underprepared national infrastructures.

In light of the fallout from the humanitarian emergency, the present article examines important considerations for designing MH interventions specific to Afghan^1^ women (AW) resettled in the U.S.; however, with so little time having elapsed since the evacuations, this article addresses all AW “resettled” (for lack of a better term) in the U.S. since 2001, so long as they underwent some manner of formal processing. It will be critical to reevaluate understandings about clinical work with AW given: (1) the limited health and MH support that persons resettled into the U.S. receive; (2) the disjointed health systems with which they interact; (3) the high baseline stress levels with which AW present (21); and (4) the acute impacts of displacement trauma.

Specifically, the article synthesizes historical, social and environmental factors in the country of origin as well as in host county contexts, assessing their impact on AW's potential receptivity to aspects of Western-derived interventions (22–26), as these are the primary modalities available within the wider U.S. healthcare system. Informed by anthropological and sociological perspectives, where knowledge gaps are identified, considerations arising from the literature are provided in order to support the development of structured, trauma-focused interventions.

There is a global “thirst for new disciplinary knowledge and practices that are grounded in equity.” (27) To address this need for actionable information, this article examines both clinical literature and interdisciplinary sources, providing insight into the documented experiences of this population. These works are further supported by the findings of clinical and ethnographic work with other displaced groups. The author complements this research by drawing from her experience in direct clinical and public health work with displaced populations, including survivors of torture and targeted violence (TTV), as well as experience obtained through national and international bodies with a focus on refugee affairs. The author has also been directly involved in the international humanitarian response to the military withdrawal, as well as in national systems and policy alignment within the U.S. so as to better serve incoming Afghan families.

## 2. Materials and methods

Given the lack of literature on clinical interventions for Afghans, let alone for AW (28, 29), the information synthesized is drawn primarily from scholarship in the fields of psychology, psychiatry, social work, medical anthropology, nursing, international relations, and other research shedding light on historical and sociopolitical aspects of life in Afghanistan prior to the onset of the 2021 humanitarian crisis. Sources were drawn from English language peer reviewed journals and book chapters, and other scholarly works, such as dissertations, as well as U.S. government, non-governmental, and inter-governmental documents and sources published prior to 13 December 2022. While the article also includes some works published prior to the U.S. invasion of Afghanistan, the bulk of the literature consulted was produced after 2001.

A search was conducted to identify literature on MH within displaced populations that maintained a specific focus on Afghans. Additionally, while the worldwide literature on displaced Afghans is considered, the clinical content of this article is informed in large part by studies and fieldwork conducted with Afghan adults resettled in high-income Western countries, such as Australia, the United States, and several European countries, among others.

For academic and NGO/IGO publications, Google and Google Scholar search engines were used to identify articles containing combinations of the following terms: “Afghan” or “Afghanistan” with “women” “clinical”, “mental health”, “trauma”, “health”, “health system”, “PTSD”, “Post-Traumatic Stress Disorder”, “anthropology”, “medical anthropology”, “BPHS”, “Basic Package of Health Services”, and related terms. Articles which only included Afghans in aggregate datasets without further discussion were excluded from this core literature, though they may have been included in the body of supplemental literature. The search resulted in 97 academic articles or IGO/NGO sources explicitly concerning Afghan MH, physical health, public health or sociopolitical aspects of Afghan society with the potential to impact family systems or MH outcomes for women. Eight similarly oriented non-NGO/non-UN news articles were also referenced as historical evidence, as well as two sources issued by the Afghan government.

To complement this core literature about Afghans or Afghanistan, additional materials included content on: “multicultural/intercultural mental health”; research on MH within other displaced populations; global and U.S. resettlement operations; therapeutic modalities; familial and gender-based violence (GBV) within other south/central Asian family systems; as well as other topics as necessary. A total of 210 materials were synthesized for this article.

The author employed a grounded theory approach for analysis and categorization of the collected materials. A number of broad categories emerged, including: literature explicitly discussing applied interventions; literature containing clinical information, but not extensively discussing applied interventions; anthropological sources; and primary sources such as news articles or government documents. Some works combined multiple elements. Emerging themes across these various categories included, but were not limited to: Afghan family dynamics and social constraints on women in the country of origin; other pre-migration environmental factors; health-seeking behaviors in a variety of contexts; relevant clinical presentations in both the county of origin, as well as in host country contexts; and interventions addressing trauma sequelae.

## 3. Literature review

### 3.1. Implications of educational variance in the country of origin

While potentially not capturing all relevant studies, a disproportionate gap in the literature is identified in that currently, publicly available research on trauma-focused interventions validated for or specifically tailored to displaced AW are almost entirely lacking, with the exception of first-line programming or other “low intensity” (30, 31) interventions. Those interventions that have been assessed for suitability for Afghans have been studied primarily with male participants [e.g., (31–34)], who inhabit a separate sphere of social expectations and constraints (35) within Afghan society. The limited literature focusing on females therefore constrains the theoretical grounding from which researchers can draw in order to develop evidence-based interventions able to meet the needs of displaced AW.

Tribe, region of origin, individual family dynamics, as well as socioeconomics and related educational levels all play an extensive role in the degree to which AW are subjected to patriarchal constraints and limited social and spatial mobility (36). In terms of education, while low relative to the overall population and very low compared to international standards, the number of women graduating from tertiary education in Afghanistan during the years of 2016–2020 (Table 1) was nonnegligible (37, 38). Furthermore, Afghanistan has traditionally been highly pluralistic (18, 20, 39, 40), and political opinions within the country therefore differ. As socioeconomic levels can often have significant impact on a person's outlook and life outcomes (41), conceptualizations of women's rights within families of higher educational attainment may vary, often differing from the prevailing norms (42, 43).

**Table 1:** Tertiary education statistics for Afghanistan 2016–2020.

Year	Unit	Students enrolled in higher education institutions at the beginning of academic year			Students graduating from higher education institutions		
		Female	Male	Total	Female	Male	Total
**2016**	Total	41,041	141,303	182,344	5,865	22,291	28,156
	Percent	22.5	77.5		21	79	
**2017**	Total	44,692	139,930	184,622	7,218	25,551	32,769
	Percent	24.2	75.8		22	78	
**2018**	Total	49,071	136,954	186,025	9,439^a^	30,480^a^	39,919^a^
	Percent	26.4	73.6		23.6	76.4	
**2019**	Total	54,861	142,386	197,247	9,937	29,687	39,624
	Percent	28	72		25	75	
**2020**	Total	62,730	142,750	205,480	960	8,955	9,915
	Percent	31	69		9.7	90.3	

Table 1 is adapted and consolidated from similar charts from the *2018–2019 Afghan Statistical Yearbook* (37) and the *2020–2021 Afghan Statistical Yearboo*k (38) on national tertiary education by gender. For the 2018–2019 report, the tables referenced are Tables 4–2 (total students) and 4–6 (graduates), and for the 2020–21 report, the tables referenced are Tables 4–20 (total students) and 4–24 (graduates). The data from both reports differ slightly, therefore wherever indicated, the more recent 2020–2021 data prevail.

^a^ Indicates that the data are from the 2020–2021 Afghan Statistical Yearbook (38).

Indeed, despite the well-documented restrictions on women's education in Afghanistan, some families support the education of their female members (44), and many Afghan activists, both male and female, have risked their lives to advance equitable access to justice and education (45–47), revealing a country with a greater diversity of ideological orientations than popular discourse might suggest. As identity is multifactorial, and as attitudes on education vary, scholars and clinicians are urged to avoid making unverified assumptions (48–50).

Indeed, the divide between the experiences of women with high educational attainment and those with little or no schooling is stark enough that Shin, et al. (51) propose that “specific support and interventions are needed for two risk groups in Afghanistan: women with many years of education, and women with no education.” This consideration is also reinforced by the findings of clinicians working with clients from Arabic-speaking countries, since “educational level is traditionally one of the central variables to control in neuropsychology” (41).

Still, while educated women may theoretically have better access to social resources upon resettlement than their less educated counterparts, the difficulties facing most AW, even those from families of relatively higher socio-economic status, should not be minimized, since most would still endorse significant life challenges and gender-based concerns (39, 42). Therefore, to better sensitize practitioners to the wide range of obstacles that AW face, this article maintains an enhanced focus on those originating from family systems where access to education and other social resources has been encumbered due to social constraints, including GBV and familial violence (FV).

### 3.2. Social constraints in Afghan society

The individualistic freedoms that inform Western concepts of human rights are often used as a barometer for gauging societal health (52); however, setting aside arguments of individualism vs. collectivism with reference to rights, women worldwide face numerous challenges to achieving gender equity beyond the restriction of their personal autonomy (53, 54). Still, by most human rights standards, as well as by international consensus, Afghanistan has very poor accountability in the protection of women and minorities, despite language to this effect in the constitution and updated penal codes (55–57).

Numerous scholars [e.g., (36, 55, 58)] have explored AW's understandings around the implications of both patriarchy and poverty on their lives, and in particular, on their access to social and material resources. Often highlighted is how the social attitudes that enable these oppressive patterns are in direct contradiction to the Afghan constitution and the rhetoric of the Karzai regime^2^ around the improvement of women's status in the region, as well as related laws aiming to present an image of a previously globalizing Afghanistan.

Public discourse on women's rights in pre-2021 Afghanistan was highly constrained, since authorities were largely reticent to engage in sustained, public, and collective action that would lift the stigma around discussing the violation of women's rights and bodies (8), as well as the reporting of gender-based crimes (56). Moreover, since crimes implicating a woman's honor could have repercussions for the family's reputation and even safety (44), bottom-up pressure on the government had been slow to arise, and when present, had been further compromised by the meddling of government officials and aristocrats (8, 56).

Add to this social imbalance protracted conflict, whether between warring domestic factions or foreign powers, and the likelihood for an Afghan woman to emerge from her social context without some major risk factor for psychological distress becomes distant; however, scholars argue that care needs to be taken to avoid further othering women by oversimplifying their suffering or minimizing their agency given that their narratives have become “rhetorically useful,” (8) already repeatedly co-opted to legitimize dramatic political shifts (8, 21, 58).

### 3.3. War, familial fragmentation and intergenerational trauma

Worldwide political instability is liable to lead to an increasing number of displaced persons, many of whom will contend with the impacts of trauma sequelae (60); Furthermore, crises may also have ongoing impacts on subsequent generations. Schaal, et al. (61) use the case of the Rwandan genocide to examine intergenerational post-traumatic stress disorder (PTSD) in countries with longstanding conflict, including Afghanistan. Their work on Rwanda reveals a conflict that eradicated more than 10% of the country's population and that left only 8% of households without the loss of a family member by “violent death.” They found that cumulative stressors and poor physical health were the strongest predictors of a PTSD diagnosis among Rwandans, who, like Afghans attempting to recover from chronic wartime trauma, may struggle with issues of complex grief.

While Afghanistan has not suffered a genocide on the scale of the Rwandan genocide (the slower-paced trend of minority persecution notwithstanding) (1), both societies share similar societal scars in that almost every household has been touched by "violent death” and that for years, the social and physical environments were characterized by unremitting chaos and fear (61). The conflicts in Afghanistan also left many orphans and widows assuming roles as heads of household within a distinctly patriarchal system, with Omidian and Miller (21) noting that 37% of the women they surveyed in the early 2000s were widows, while none of the men surveyed were widowers.

The violence inflicted on Afghan society, whether by domestic forces or foreign powers, has the distinct potential to create lingering inter-generational trauma (62). Moreover, it has led to forcible displacement and community disruption through social reorganization (61, 63), and cycles of distress are perpetuated in large part by the fact that caregiver MH is correlated with negative MH outcomes in children and adolescents (64, 65). In light of this most recent emergency, Qamar et al. (66) have declared the trauma potential on Afghan children as “an impending catastrophe,” a sentiment supported by a prior school-based study, which found that even by 2006, in certain areas of the country, one in ten children had lost one or both parents (65).

### 3.4. Gender-based violence

Turning to the domestic sphere, the situation of AW is not wholly unique in the world, since women are subject to disproportionate levels of systemic, intimate partner, and familial violence as compared to men, with one in three women globally having been subjected to violence (67). While women in other Islamic contexts (e.g., Iraq and Syria) may be said to face similar challenges (68, 69), the Afghan milieu has a unique combination of social, geopolitical, and tribally influenced religious interpretations (70) that maintain an elevated potential for adverse health outcomes among women. Not least among these factors are the length of military occupation and complete foreign dominance over national systems, which, from the time of Soviet occupation onwards, have fomented social volatility (21). Additionally, women in general have been observed to have poorer MH outcomes from displacement than men (71).

Rasmussen et al. observe that gender greatly circumscribes experiences in Afghanistan since “men and women inhabit different social and emotional spaces… with downstream implications for psychosocial and physiological well-being.” Though some women may well lead fulfilling lives, far more would describe some variety of imbalance between the genders, regardless of whether it is framed in familial, political, social, spatial, or religious terms (36). FV, including IPV, impacts a disproportionately high number of AW.^3^ Though natal families can sometimes confer protection from gender-based abuses (73, 42), married Afghan women may be mistreated not only by their husbands, but also by their in-laws in manners similar to those observed in other Central Asian and subcontinental regions (74, 75), as well as in various areas of the broader Middle East (76, 77). Women who do not wish to conform to the obligations imposed on them have little social or legal recourse, as laws against FV are not consistently enforced, and since household conflict is typically viewed as a private, domestic matter in which it would be shameful for outside parties to intervene. This heightened perception of FV as a private matter is seen in a variety of low-income countries and is rendered more complex due to a broader understanding of kin relationships (55, 78, 79).

Intimate partner violence (IPV) specifically poses challenges in Afghan communities, with Metheny and Stephenson (57) finding that the “acceptability of IPV in Afghanistan is widespread, with 80% of women and 72% of men in the 2015 [Afghanistan Demographic and Health Survey] agreeing physical IPV is justified.” There has also been a near-direct connection of FV to the economic insecurity and feelings of financial powerlessness experienced by males struggling to maintain ever-scarce employment, and feelings of inadequacy may subsequently be displaced onto female relatives (36, 55). Often exacerbated by substance dependence (80), multiple risk factors have rendered a generation of Afghan men increasingly incapacitated, leaving women to assume greater responsibilities. As a result of these economic hardships, many Afghan women engage in significant amounts of domestic labor, while also engaging in some form of income generation, though these activities are frequently limited in scope to preserve their dignity as married women (36, 55). Moreover, the gendered agendas of foreign intervention have added to the friction between the male and female spheres (45, 81) in manners reminiscent of the “continuums of violence” described by Yadav et al. (82).

All of these factors combine to create a highly volatile environment in which women must negotiate numerous expectations. Given the weight of expectations placed on them, many women feel resigned to their roles, even if they think these dynamics unjust (36). Many also perceive their struggles to be intractable (83) and feelings of helplessness and anger were further ingrained by inadequate and complicit lack of policing, as well as the lack of enforcement of protections for women (36, 55, 57, 84). The intersection of low educational attainment and female sex can thus lead to high psychological and physical morbidity in Afghanistan (42, 85). Women, especially those situated in the more resource-scarce tribal regions, are often born into poverty and restricted to minimal, if any, schooling, and many are married quite young with the intention of protecting them multiple outside dangers, economic insecurities, or perceived threats to family honor (86).

### 3.5. Prioritization of daily functioning

While not all AW endorse MH consequences stemming from these adversities, a great number of women from a variety of backgrounds would (36, 55), and many women who have experienced conflict trauma and displacement, might cite their daily stressors as being more significant (21, 24, 28, 42, 87, 88). This is supported throughout the broader displacement MH literature (DMHL) which posits that the trauma symptoms that many resettled women face could be ascribed more to ongoing stressors than to acute conflict exposure (89). In displacement contexts, this is due in part to continuous upheaval in the host environment (48), creating a “permanent state of emergency” (90). Therefore, chronic distress, poverty, isolation, lack of access to medical care or legal services, and lack of stable food and water may act syndemically within an environment in which women's roles are already highly constrained (29, 57, 65, 85, 91, 92).

### 3.6. Afghans in resettlement contexts

#### 3.6.1. Resettlement of Afghans in the U.S.

A full discussion of U.S. resettlement mechanisms as well as the services provided to Afghans arriving to the U.S. during Operation Allies Welcome (OAW) are beyond the scope of this article. Additionally, the highly fragmented response extended well beyond traditional resettlement frameworks, involving highly heterogenous and atypical case processing (93); however, the range of formal vehicles for entry into the U.S. under OAW primarily included the Special Immigrant Visa (SIV) program (94), as well as categories of humanitarian parole and priority designations (95, 96). Some of these processes could be initiated in Afghanistan, while others required entry into a third country before processing could begin (96). Prior to OAW, Afghans resettled in the U.S. usually entered through the SIV program or as refugees.

The totality of intersecting immigration mechanisms used during OAW has the potential to create trauma on a population-level, especially in light of the severe manpower, disciplinary, and service gaps that exist for supporting these complex cases upon arrival (97). Among these gaps is the provision of MH services, which has been identified as a high priority by the Office of Refugee Resettlement (ORR), the federal entity charged with administering post-arrival services, among other responsibilities (98). Currently, policy around access to MH supports continues to change rapidly, and flexibilities introduced by the U.S. government (97, 99) currently allow OAW beneficiaries greater access to an increasing number of programs under the purview of ORR (100).

Future research is well-advised in order to assess the impact of these various mechanisms, processing durations, and time spent in third countries, as well as the quality of post-arrival services received; Regardless of the mechanism used, commonalities exist between these cases, including: (1) the fact that resettled families necessarily left Afghanistan; (2) many of these mechanisms involve arduous processing; and (3) many families eventually became eligible for some sort of supportive services (100, 101).

In addition to cursory screening by designated refugee health clinics, formally resettled refugees are connected to community health systems as part of reception and placement services or intensive case management programs, such as Preferred Communities (if eligible) (102). Nevertheless, even if enrolled into intensive medical case management, these programs frequently support clients in making connections to health systems not specialized in migration medicine.

#### 3.6.2. Clinical presentations

Afghan Women who arrived to the U.S. or other high income countries prior to the 2021 humanitarian crisis typically presented with high incidences of physical ailments, including chronic illness, hypertension, difficulty sleeping, and dental problems (28, 29, 103), in addition to MH challenges, including depression, anxiety, and somatic concerns (28, 29, 103, 104). Breakdowns of Afghans' presenting conditions in resettled contexts consistently find elevated rates of depression, anxiety, and PTSD. For instance, a 2002 study found a 57% prevalence of depression and a 35% prevalence of PTSD diagnosis among Afghan refugees in the Netherlands (105), though other studies have found the prevalence of PTSD or severe psychological distress to be much higher, from 46% (106) through to 100% (104, 106), dependent on age, and potentially other factors, such as education. Barriers may also include linguistic challenges (29, 104). Though some women arriving under OAW will be highly literate in English or another NATO country language, the majority will lack the tools for self-advocacy without the assistance of an interpreter, leading to possible distress and potentially complicating health evaluations (29, 104).

#### 3.6.3. Challenges to understanding health-seeking behavior in resettlement contexts

Literature on refugees' social and medical access reveals that regardless of an individual's background, resettlement to a new country does not necessarily translate to improved opportunities or mental health within the new context (107–109), as resettlement can introduce “non-income” dimensions of disadvantage (92). Afghan Women have also, at times, been subjected to even greater seclusion upon displacement due to familial concerns about the unfamiliar environment (110).

However, existing findings on Afghan's health-seeking behavior are somewhat inconsistent with the findings of other works on the relationships between resettled persons' education, language proficiency, and health seeking behavior. For example, a study by Gerritsen et al. (111) that included resettled Afghans revealed no association between education and use of healthcare services. Furthermore, Tomasi et al. (112) demonstrate that female sex and older age are positively correlated with health-seeking behavior among Afghans, but indicators of acculturation, such as not needing interpretation assistance, had no association with health-seeking, leading the authors to posit that, "it may be plausible in our study that those refugees who were less acculturated were experiencing poorer mental health and, thus, as suggested in Anderson's health care utilisation model, had a greater need for professional help.” Moreover, Afghans have also been shown to be receptive to pharmacological interventions under certain circumstances, but findings on attitudes vary (24, 106, 111). Therefore, some scholars note that inflexibly holding assumptions about the meaning and implications of health literacy, as well as health-seeking behavior, can be perceived as dismissive and may create oppositional dynamics where perhaps none were warranted (28, 29, 83, 113).

Gender concerns also rise to the fore in health seeking, since several studies note that both in Afghanistan and in resettled contexts, women may be dissuaded from using the available health and service systems due to the number of men with whom they may need to interface prior to obtaining help from a female provider (19, 29, 103, 114–118). Other gender dynamics may also come into play at point of care, such as husbands insisting on translating for their wives during assessments (119).

In Western medical contexts, clients in the general population who are exhibiting post-traumatic symptomatology may be referred for trauma-focused therapy. Services would then likely be delivered through a number of possible modalities, ranging from approaches based in Cognitive Behavioral Therapy (CBT) (120, 121) to more somatically or processing-oriented modalities^4^, such as cognitive processing therapy (CPT) (124), eye movement desensitization and reprocessing (EMDR) (125, 126), newer approaches, such as accelerated resolution therapy (ART) (127), or similar treatments.

Though smaller-scale programming might exist in certain pockets, on the whole, interventions offered to resettled persons through everyday community MH centers are unlikely to have been tailored to the needs of specific foreign-born populations to any significant degree, especially given a global lack of trained migration health professionals (128). This gives rise to concern, since referring a client for MH services without first assessing for the modality's compatibility carries the potential for retraumatization (25), and unpleasant first encounters or triggering stimuli for survivors of TTV are liable to reduce adherence to treatment or create negative perceptions (29, 32). This lack of tailoring could constitute a confounding variable when observing service uptake rates.

Another difficulty is that providers in host countries may fail to understand clients' concerns about the perceived integrated nature of their ailments (129). This is particularly important with reference to somatization, as well as for survivors of TTV given that the physical consequences of violence can serve as reminders of the humiliations endured (31, 60, 130–132). For a client, selective attention to certain symptoms over others may lead to feelings of being dismissed or unheard, with Kaiser et al. (129) noting that “idioms of distress” may become reduced to “psychiatric categories” (129), especially in the hands of less experienced practitioners. On this essentialism, they further observe that there is “potential harm associated with reducing these idioms to a single, closest psychiatric diagnosis.”

Consequently, researchers assessing service uptake would have to first ensure fewer barriers to service, and they should also provide substantial, proactive education in a format acceptable to the client about how to access the health system in question before measuring willingness to engage with available services. Despite these knowledge gaps, there exist a few interventions specifically tested for compatibility with Afghan norms. These studies could help researchers devise interventions that would help mitigate the challenges faced when high numbers of arrivals threaten to overwhelm already inagile healthcare systems (30–32, 34, 133–135).

### 3.7. Prior clinical interventions with Afghans

Currently, the literature on clinical approaches for working with Afghans is seriously lacking (51), and, at times, contradictory. Most conspicuous is the absence of rigorous, evidence-based and trauma-focused interventions appropriate and specifically designed for their psychosocial needs. Firstly, PTSD as defined by the DSM (136), has been said to have good construct validity in that it aligns with the symptom clusters reported by Afghans having experienced trauma, and in that it has a positive, yet “modest” correlation with war-time events (137); however, it has been postulated that it has poor clinical utility (137, 138). As Miller et al. note, “traumatic stress was marginally more highly correlated with depression, and significantly more highly correlated with generally [sic] distress, than it was with PTSD.” They also observe that “… cumulative lifetime challenges lead to sadness being among the most salient experiences in high-conflict environments.” (137)

Clinical assessments with Afghans may also be affected by “‘gender-appropriate’ expressions of emotion” (137), and some individuals may feel that divulging too much of their experiences might burden others suffering from similar issues, while for others, concerns of modesty may restrict disclosure to clinicians (29, 139, 140). Therefore, women often turn to confidants within their social circle, including the approximately 20% of women who seek support for IPV by consulting with extended family (28, 29, 57, 137). To this end, Ahmad, et al. (141) note, “The high prevalence of PTSD, its association with social support and self-rated health are important issues to be considered for refugee resettlement programs.” They also add that this communal orientation might be particularly true of client systems that “originate from communities with strong social bonds and ‘collectivist’ values” (141). In terms of other coping strategies, studies have found high numbers of respondents attesting that their greatest comfort lies in religion, while other studies demonstrate that women from Islamic contexts may attribute their trials the supernatural (24, 29, 51, 106, 138, 142–144).

With reference to applicable interventions, there exist benign approaches designed for emergency contexts, but which are malleable enough for adaptation to specific populations. Among the more well-known of these is the World Health Organizations' Problem Management Plus (PM+), which can be adapted for individuals or groups (145–147); however, a limitation of this and similar formats is that sustained intervention is rarely feasible in camps or other settings with severe challenges. Therefore, approaches such as the PM+ do not target the trauma itself to any great degree and instead focus mainly on quickly building problem management skills and self-regulation techniques to better manage feelings of overwhelm (30, 147). Nevertheless, scholars such as Knefel et al. (133) support adaptations of the PM+ for Afghans, with Knefel et al. finding that the model is positively received by MH professionals. Importantly, though, several of the clinicians in their study reported that they found the intervention too limiting and that those “with more work experience with refugees were less likely to use the interventions than those with less experience” (133). Models of this sort might therefore serve more as introductory training for clinicians rather than robust, stand-alone treatments. Still, the perspectives afforded by the PM+ and similar systems could help supplement more intensive trauma-focused therapies.

As for preferred intervention formats, displaced persons expressed appreciation for group-based models in a study by Mitschke et al. (148). Other studies have investigated modalities falling outside of the standard dyadic talk-therapy format with Afghans^5^^,^^6^ [e.g., (31–34, 134, 135, 149)]. Various works by Kananian and colleagues, for example, provide an insightful overview of trauma-informed approaches for working with resettled Afghan and Iranian males in group settings. In addition to employing some of Hinton's work on Culturally Adapted Cognitive Behavioral Therapy (150), Kananian et al's collective works (31–33)^7^ are noteworthy for: delivery of services in the target language; the articulation of MH concepts in a manner congruent with participants' conceptualizations of health; the centrality of somatic concerns to the project; sensitivity to TTV history; the consideration of logistical challenges (which they reduced through schedule optimization); an emphasis on client psychoeducation; and the real-time incorporation of client feedback.

In other work, Droždek, et al. (34) conducted a ten-year study on a program for Iranian as well as Afghan refugees and asylum seekers who had survived torture and had been resettled in the Netherlands. The participants were enrolled in trauma-focused group therapy administered within several different intensive day program regimens. The study compared the efficacy of different treatment intensities between the various groups, as measured by the number of weekly sessions. The major findings of the work were that intensive, yet less expensive day treatment programs decreased symptoms greatly as compared to the control group, and that “more intensive and expensive inpatient treatments are not necessarily more efficacious than the less expensive day hospital alternatives” (34). These findings are reinforced by a study with non-Afghan refugees that observes that participants preferred more frequent outpatient group sessions to meet their needs (148).

Importantly, Droždek et al. (34) found that exposure to others' trauma histories within a group setting was not found to have any harmful effect despite the concern about secondary traumatization, nor did it cause any group members to drop out, since, as the authors speculate, the universality of these narratives among the members appeared to create social cohesion (34). The importance of community may also be reflected in the work of Griner and Smith (152), who concluded that “MH programs targeting culturally homogenous client groups were four times more effective than those targeting culturally heterogeneous client groups” (152). Though it offers an interesting perspective, a limitation of Droždek et al.'s work is the inclusion of only men, since the intersectionality of women's experiences (21, 24) may require specific attention. This is particularly salient for widows, who have been reported as having the highest levels of stress within Afghan society (24).

In other interventions, Berdondini et al. (135) worked alongside both male and female Afghan MH professionals to develop a cross-institutional program in which the Afghan clinicians themselves served as clients. The colleagues then exchanged perspectives arising from the experience, deliberating about how to best address (or not address) taboo topics, as well as how to draw out information from clients without seriously violating norms (135). Their work highlighted the potential incompatibility of approaches primarily dependent on frank speech with Afghan values. In their assessment, not only are certain subjects and emotions entirely taboo in the Afghan context, but too candid of expression of familial conflict, personal worries, or insecurities also violates social convention within a highly collectivist and interconnected society (135).

Ultimately, Berdondini et al. (135) concluded that psychodrama, molding, and other activity-based methods for processing interpersonal conflicts could be much sounder than questioning or probing some issues directly. They write, “this methodology proved it was possible, in a culture where clients cannot explicitly disclose their worries and concerns, to run therapeutic sessions that are non-diagnostic, non-directive, [and] very respectful of client's needs…” (135). One might thus be able to regard Berdondini et al. (135) and Drozdek et al.'s (34) studies of group therapy as potentially at odds in some respects, especially with regards to candor; however, results may be dependent on the topics that emerge when debriefing members' traumatic experiences. Nevertheless, a possible takeaway of Berdondini et al. (135) is that group-based approaches that too overtly name feelings or that too explicitly detail personal matters might therefore be considered inappropriate, or at minimum, they might warrant caution.

While not a clinical intervention in itself, Ramezani et al. (153) offer a noteworthy resource on neuropsychological assessments with Afghans. The authors offer a brief review of historical and cultural considerations, as well as approaches deemed potentially generalizable to the wider Afghan population. Critically, they offer commentary regarding the utility of certain diagnostic tools, as well as an appendix of applicable instruments, which constitutes a highly valuable contribution of this work. The text also addresses Afghans' tendency to attribute distress to supernatural causes, as well as observations on male and female dynamics during clinical interviews, such as deferential behavior and situations in which it might be acceptable for a woman to speak on behalf of a male family member to preserve his dignity.^8^

Notably, future interventions could also integrate the principles of a growing movement that emphasizes the inclusion of restorative justice within trauma-based work (154). Literature on such approaches, as well as literature on “ecological” models, emphasize the importance of simultaneously attending to individual, as well as societal health (104, 155). The Center for Victims of Torture (CVT) has worked on one such initiative, and in addition to providing other interventions as appropriate, CVT and its partners offer survivors the opportunity to share their torture narratives in a manner which not only carries therapeutic utility for the interviewee, but which also augments the global documentation of mass human rights abuses (156, 157).

In so doing, “testimonial therapies” (26) afford survivors the opportunity to articulate visions of what proper justice might entail, and reports compiled from the aggregate data could be made available to entities with the ability to act upon these insights (156, 157). An emphasis on concepts of justice when working with Afghans is also supported by the work of Lopes Cardozo, et al. (84), who found that “a substantial number of respondents reported that revenge would not be necessary if a justice system existed. Establishment (or reestablishment) of a justice system may be essential for postwar societies to decrease feelings of hatred and revenge.” (84) Though testimonial therapies would not constitute formal redress, the fields of restorative and transitional justice continue to evolve, enhancing the meanings and responsibilities inherent to working with individuals having sustained trauma or TTV.

## 4. Results

An analysis of existing work on Afghan MH (as supplemented by the broader DMHL) highlights several areas requiring further investment in order to better support to providers interfacing with displaced AW. This includes key considerations in the design of comprehensive, structured, and trauma-focused interventions. Findings include: the need to supply practitioners with actionable content; the need to evaluate the applicability of mental health work with other displaced populations; the need to improve the dimensionality (158) of medical education and studies on displacement mental health; the need to better understand Afghan women's health-seeking behavior; the need to reevaluate CBT as a dominant modality; the need to explore new obstacles to feasibility; the need to continue working towards “community-engaged scholarship” through ethnography and participatory research; the need to support resettled survivors of familial violence; considerations regarding the integration of religion; considerations regarding employment or educational programs; considerations for reinforcing identified modalities through an enhanced focus on restorative justice and social impact; and considerations regarding the generalizability of resulting interventions.

## 5. Discussion

### 5.1. Actionable content for clinicians

Answering the call for equitable MH models (27, 183), significant progress has been made over the last couple of decades in fostering practitioner awareness of differing conceptualizations of MH across cultural contexts; however, the terms “multicultural” or “intercultural mental health” might, at first, seem to serve as an umbrella term covering a breadth of studies on a variety of discrete social systems. It may, therefore, be helpful to disambiguate “multicultural/inter-cultural MH” as an overall approach, vs. collective bodies of clinical work with specific populations. Though there are varying definitions, the driving force behind the field of “multicultural health”, in broad form, could be said to emphasize the exploration of philosophical frameworks, competencies, and methodologies (50, 159, 160) characterized by “reflective self-awareness” (161) and inquiry, among other tenets. To this end, Sabnani et al. (162) write “The single most significant advancement in cross-cultural counseling practice and research in the last decade centers on the salience of both the client's and the counselor's racial identity development to the cross-cultural encounter.” This same inquisitive ethos would be critical to working with any population, including Afghans; however, literature of this variety does not always easily translate into actionable approaches that health workers could readily employ. Overall, actionable translation of the aggregate findings of clinical and anthropological investigations might be more helpful in this regard.

### 5.2. Applicability of prior mental health work with other displaced populations

In the absence of actionable frameworks, treatment centered on ill-fitting diagnostic models can be alienating to the intended beneficiaries and may therefore have limited, if not poor viability (26, 50). They may also be perceived as invasive among individuals who have already been subjected to severe incursion on their bodily autonomy and rights throughout their lives (28, 29). Furthermore, few programs are directly shaped by the voices and preferences of participants themselves, reflecting a wider problem within health system and social service development (163).

Additionally, the studies discussed in this article have mostly focused on group formats. Though dyadic formats may not necessarily be contraindicated for AW, as close relationships to a provider can have rehabilitative properties through the “therapeutic use of self” (164, 165), some of the points raised by the literature around the viability of certain approaches would call into question many of the standard, one-on-one talk-based interventions commonly used within the wider U.S. MH environment. Questions emerge around client preferences for format and level of disclosure, as well as gender and other power differentials described in the studies mentioned. Also, their findings may be influenced by the fact that group-based approaches may have been preferentially researched due to greater feasibility and efficiency. Future research may therefore wish to verify preferences surrounding group vs. dyadic formats.

Moreover, when faced with high numbers of individuals from a relatively new or underserved population, the response by the provider community might be to turn to the closest body of evidence-based information. This may include consulting studies performed with those populations most closely resembling the socio-linguistic backgrounds of the clients in question if sufficient literature on that group is lacking, and some clinicians with less training in working with the displaced may presume Arabic-speaking groups to be a suitable proxy for Afghans.

The collapse of the Syrian state has yielded an abundance of literature on displaced Syrians' MH (166), including related interventions (167). Yet, however useful the resulting literature may be for understanding "refugee mental health" as a discipline, challenges exist in applying these learnings to the Afghan context. Sociolinguistics aside, practitioners must consider that in exceedingly broad strokes, the economic and educational contexts of Syria (in which, prior to the war, 93% of the general population had or was in the process of attaining basic education) were much higher as compared to those in Afghanistan. During the 2012/2013 academic year, approximately 659,394 Syrians were enrolled in higher education institutions, whereas in 2015 Afghanistan, the adult literacy rate was 38%. This is a level substantially lower than the international average (84%), and also lower than the averages of neighboring Iran (87%) and Pakistan (56%) (168, 169).

Another aspect to note is the impact of terrorism and foreign dominance over the political landscape. While ISIS eventually rose to power to occupy the vacuum created in the Levant following the collapse of Syria, the Afghan situation differs significantly in that known terrorist groups have held considerable sway over geopolitical realities for decades, allowing for widespread violence against women (170, 171) and minorities (172). It should also be noted that for approximately twenty years, the development and maintenance of Afghanistan's national infrastructure had been largely dependent on U.S. and NATO country presence, resulting in many families having ties to global powers –ties which were abruptly severed following U.S. withdrawal. Though Syrians have expressed feelings of betrayal by the international community (173), the impacts of these long-standing relationships, dependencies, and any related feelings of betrayal (42), may differ among Afghans and Syrians. In this respect, the literature on Iraqi SIV recipients could possibly demonstrate some similarities, though here too, there would be reason to question wholesale applicability (106).

Other concerns arise regarding the choice of modality. Many of the interventions in DMHL are also limited by factors such as an overemphasis on mindfulness, other benign grounding or self-regulation techniques, and “expressive therapies” (26), such as art therapy and photovoice (174). The collective scholarship on MH within displaced populations that does engage in more intensive clinical work demonstrates an overreliance on modalities based in Cognitive Behavioral Therapy (CBT) (26). While researchers would need to validate the suitability of more intensive therapies targeting hyperarousal of the autonomic nervous system for this population, in the general population, therapies such as EMDR and exposure therapy have at times been found to have equal if not greater effect on traumatic stress symptoms than CBT (175, 176). Moreover, these approaches have been shown to help with complex grief, which affects many AW (177). Attention to chronic hyperarousal could thus offer women much needed relief from trauma sequelae and may support the increased daily functioning which Afghans and other persons suffering from Disorders of Extreme Stress Not Otherwise Specified (DESNOS) have been seen to prioritize (178, 179). Additionally, these approaches are often less verbally reliant than CBT, mitigating interpretation challenges, as well as discomfort with addressing socially unacceptable topics. Possibly assuaging concerns about non-adherence, some studies have shown that modalities of this sort do not necessarily lead to increased attrition in the general population, with Hembree et al. (180) finding “no difference in dropout rates among exposure therapy, cognitive therapy, stress inoculation training, and EMDR.” It is possible that the quality of preparatory psychoeducation may factor into client receptivity.

Though the increased global interest in “refugee mental health” could be seen as a net positive, the field on the whole still requires significant investment. Along with associated benefits, intense surges of interest in a given crisis may also cause harm in that entities not grounded in the practice of working with impacted populations can reinforce hurtful paradigms and can engage in counterproductive underestimation of client-agency, especially since the broader DMHL demonstrates “a variety of methodological concerns”, as well as limited understanding of the dimensionality of trauma (26). Many studies that engage in more detailed discussion of trauma also focus on psychometrics with little disambiguation of precursors beyond the well-established traumatogenic experiences that refugees have, by most definitions (181), lived thorough, including persecution, exposure to conflict, resettlement, and post-resettlement frustrations (26, 106). This non-specific characterization of trauma leaves the clinician unfamiliar with working with the displaced without many consolidated, evidence-based, and actionable resources for deconstructing challenging client presentations. In remedying gaps around trauma-focused work with Afghans (and other displaced populations), practitioners are encouraged to continue building on the existing literature by investing in works that move beyond pure psychometrics, through to a more inquiry-based ethos combined with actionable content.

### 5.3. Improved understanding of Afghan women's health-seeking behavior

Numerous research teams over a wide array of destination countries speak of “low MH literacy” as a strong predictor of poor health-seeking behavior among refugees and asylum seekers (132, 183), including Afghans (184, 185); however, while many scholars account for the contributions of daily obstacles to lower service uptake, practitioners should still be cautious in taking assertions about health-seeking behaviors at face value, as research demonstrates that system use could vary according to logistical variables (107, 185).

In sharing their perspectives on health interactions, AW and their families have reported benefiting from the support of healthcare navigators or community supports, demonstrating a desire to engage with systems (29, 186). While their desire to engage with services has been documented through such works, the health-seeking behaviors of AW must also be reevaluated, as the absence of compatible services may lead to decreased engagement. Firstly, ascribing low MH literacy to a displaced person without sufficient investigation of context, especially if the client is unfamiliar with the host country language or systems, may accurately describe a client's unfamiliarity with biological and psychological principles; however, incorrect meaning can be ascribed to women's decreased pursuit of medical resources when reduced to statistics without sufficient qualitative investigation, especially since poor uptake may instead stem from repeated failure to reach adequate help, or the inability to obtain it through the prescribed channels (28, 29).

Moreover, women may already identify their grief as being problematic since it leads to decreased functioning or the inability to fulfill responsibilities (21). They may also be aware of the situational etiology of their symptoms and may be familiar with formal or informal means of alleviation, and they may also take steps to restore their psychological health *via* their physical health or vice versa (29, 106, 189). Such agency could constitute a reasonable level of MH literacy, whether or not it adheres to the Western clinical model. Researchers will therefore need to better capture these client understandings, and practitioners should know how to work with and redirect such clients as needed.

Nevertheless, the surge of Afghan arrivals to host countries presents a unique opportunity to reevaluate “risk and mitigating factors for mental illness” (84), personal coping strategies (187), as well as the “societal and individual” factors (188) affecting resettled women's health-seeking dynamics. This would be an especially important undertaking for AW, who have traditionally been marginalized in both society and clinical research, despite ethnographic studies finding Afghan communities “eager for culturally adapted interventions“ (103). This is further supported by findings noting a high level of trust placed in the ”biomedical community” (inclusive of psychologists and psychiatrists), as well as their view that that certain conditions can be ameliorated with their help (106, 189).

### 5.4. Isolating the impact of individual stressors

The aggregate literature on Afghan family systems indicates a variety of pressures with which women contend, and ongoing stressors are often endorsed as being more impactful than emotional concerns (21). Contributors to MH presentations include social constraints, such as societal tolerance of GBV and FV, as well as logistical barriers, such as lack of familiarity with health systems, distance from health centers, poverty, and food and water insecurity, among other factors (85, 163). Unsurprisingly, war-related trauma also directly affects a great number of Afghan households (65, 85, 89). Though other frameworks may be developed, based on the available evidence, and building on the work of Omidian and Miller (21), the stressors most salient to AW's experiences can therefore be categorized into the following broad categories: war or conflict-related trauma; chronic/background environmental stressors not directly related to violence; FV and GBV; and resettlement trauma.

A first step in identifying promising clinical approaches could be to verify the proportionate impacts of these stressors, isolating which factors would be paramount to rapidly attenuating stress levels. One possibility would be to enroll married or previously married AW who have met the following four conditions: (1) have suffered direct, acute trauma in connection with war or conflict; (2) have been exposed to chronic, non-conflict environmental stressors; (3) have experienced FV; and (4) have been resettled. These and similar stressors could then be isolated and controlled across experimental groups. If desired, those groups addressing war/conflict-related trauma could be further divided to better isolate the effects of trauma endured prior to the 2021 withdrawal, as well as the trauma endured as a consequence of this event. Of note, given the social isolation experienced by many resettled women (29, 190), researchers could also consider controlling for increased social contact *via* interaction with personnel, even if contact is minimal. Symptom improvement could then be tracked over a number of months using the Afghan Symptom Checklist (24), the Hopkins Symptom Checklist (191), the Harvard Trauma Questionnaire (192), or other applicable instruments, and results could be analyzed *via* qualitative and quantitative methods. Other approaches could certainly be proposed, but targeted attenuation of baseline stress levels could lead to better daily functioning, which Afghans have been observed to prioritize (24, 106).

### 5.5. Examining CBT as the dominant modality

In addition to evaluating receptivity to different formats, content and intensities, researchers could explore various modalities, and sequencing of themes. For example, researchers could overlay differing subject matter with modalities directly targeting PTSD-symptoms (processing/exposure/desensitization/somatic therapies) or non-verbal modalities (91).

These approaches could then be compared to more “actionable” modalities targeting cognitively rooted symptoms, such as standard CBT. Comparative research of this sort would be insightful given that CBT is among the most frequently used modalities in research with displaced populations (26). This overrepresentation likely reflects the general palatability of CBT approaches for a variety of clients, as well as its flexibility and ease of incorporation into other methods, but it may also reflect the relatively higher barrier to clinician qualification in more expressly trauma-focused modalities. There is, therefore, significant need to explore the viability of non-CBT interventions for those desiring more intensive treatments. This sentiment is echoed by Kananian, et al. (31) who comment that CBT is appropriate for those needing, tolerating or desiring lower-intensity treatment. Stratification according to intensity is also encouraged by Panter-Brick, et al. (65) who observe that, “Emerging consensus advocates several layers of support for MH programs in emergency settings: those targeting the family and community, as well as those offering more specialist care for those in clinical need.”. Participant interest in focused mental health programming is further supported by the findings of Yaser et. al (106), who observe that participants”…viewed the use of specific psychotherapy in the treatment of PTSD favourably. The vast majority considered both' psychotherapy focusing on changing thoughts and behaviours' (84%) and 'psychotherapy focusing on the past' (86.7%) to be helpful.”

Whatever the approach, psychoeducation is advisable in the case of more intense treatment, since clients undergoing exposure-based therapies or similar treatments may question how uncomfortable stimuli and the disturbing symptoms which they elicit, such as intrusive thoughts and flashbacks, could constitute a pathway to relief. Also, populations experiencing ongoing adversity may default to avoidant coping strategies that may be correlated with PTSD (141), and intense therapies may trigger these behaviors, though as Wildt, et al. (138) write, “the direction of causality is unclear.”

Future research could also consider examining symptoms through the lens of C-PTSD, as defined by the International Classification of Diseases (ICD) (193) rather than through the Diagnostic and Statistical Manual's (DSM) framework for PTSD (136). Silove, et al. (194) observe:

… difficulties encountered in providing effective treatment relate in part to a failure to acknowledge the extent of the [Traumatic Stress (TS)] response which extends beyond core PTSD to encompass symptoms defined in ICD-11 as C-PTSD. It may be that refugees with this more general TS response which encompass difficulties in functioning and adaptation, warrant more extensive, multimodal approaches to treatment and rehabilitation which go beyond a focus on overcoming fear-related symptoms thought to underlie core PTSD.

### 5.6. Obstacles to feasibility

It must be acknowledged that given the well-documented difficulties around resettled populations' access to health resources (195), implementing certain research designs with AW, especially those from more conservative or rural families, may remain partly aspirational. Even if they may wish to participate, obstacles may be encountered, such as: (1) participants may not be sufficiently forthcoming with their concerns to be properly assessed or categorized; (2) numerous logistical barriers to accessing services, such as childcare and transportation; (3) too few women actively seeking sustained care to create a valid study (196) and (4) women being denied permission to participate by their families. Attention should also be paid to the ethnic groups to which the women belong, as well as their perceptions of other groups (48) since some may be opposed to mingling with participants of different ethnicities. Still, the minimization of barriers should remain integral component of comprehensive social services since women may be motivated to participate absent any major logistical barriers (103).

### 5.7. “Community-engaged scholarship” through ethnography and participatory research

In the face of myriad structural and ideological barriers, it is essential that health systems be prepared to interact with individuals for whom, due to multiple legacies of oppression, the present manifestations of the Western medical system might not be as desirable (50), but who nonetheless might benefit from some form of intervention. The past decade has seen a rapidly growing interest in the decolonization of international development, and by extension, the global health field's Western-centric orientations (197–199). Moreover, while it could be argued that psychiatric diagnostic labels are a useful shorthand for describing symptom clusters, studies of trauma across international contexts have raised questions over the universal utility of Western-developed constructs (24–26, 109). Kaiser et al. (129) describe differing conceptualizations of symptoms, stating, “Ethnographic research is informative here because it facilitates the identification of cultural concepts of distress that communicate the complex etiology, meaning, and response surrounding forms of suffering.” Some of these details may not be captured through works of the “quantitative research tradition” (200), so scholars increasingly advocate for the utility of medical ethnography and “community-engaged scholarship” (158) in the education of health professionals (158, 200), especially given the global lack of trained providers (183).

When designing interventions for understudied or marginalized populations, any results observed (and programs subsequently developed) should be further tailored through focus group input in order to verify compatibility with the social constraints of the new environment. Additionally, and in line with promising practices in trauma-informed participatory research (67, 201), all phases of the process should involve Afghans in both research and leadership positions so that they may shape the structure of the intervention. Furthermore, participants could have the function of optimizing the study environment so as to avoid re-traumatization, as well as establishing an atmosphere conducive to engagement. Practitioners would also need to maintain an eye toward “community-based system dynamics” for improved monitoring of feedback loops (163).

### 5.8. Afghan clinician training

While paternalism should be avoided wherever possible, a related consideration centers on how to best integrate Afghan health professionals in participatory research while still observing the highest level of medical standards. Mismahl et al. (202) have found MH provider training within the Basic Package of Health Services (BPHS) to be inconsistent, with the authors stating that, “even doctors trained in internal medicine and specializing in mental disorders within MH hospitals require training in basic neurological examination skills.” This aligns with their findings that some psychosocial workers conflated “giving advice” with having performed quality intervention. Within the BPHS, many psychosocial workers received only a year or so of specialized training (202). Therefore, research teams should be aware of these potential gaps in training rigor and potential inclinations toward directive approaches.

### 5.9. Supporting resettled survivors of familial violence

There exist important ethical considerations surrounding FV, and given the vulnerable intersection of displacement and FV, the reality may be that until the legal and social service systems of the destination countries catch up to their ideals, these systems may not be equipped to handle the specific needs of refugees experiencing FV. This is especially worrisome given the lack of literature on appropriate responses to GBV in displacement settings. This lack of informed guidance is conspicuous, and in their analysis, Asgary et al. (203) wrote, “We did not find a single article evaluating either the prevention or treatment/management of GBV and its health consequences in displaced populations that met the inclusion criteria.”

Additionally, and with the exception of cases involving minors, isolating a woman from her family system before she feels empowered to leave on her own may cause considerable hardship, as seen with South Asian women, including immigrant women, who often face many obstacles in leaving their abusers (78, 204). Resettled women may face even more obstacles given the brief time that the systems allow for resettled individuals to attain financial security, with Wachter et al. noting that, “poverty interferes with women's ability to meet basic needs, forcing survivors to prioritize housing, food, and employment over safety and well-being” (205). Therefore, assuring that abuse is no longer ongoing would render studies safer for participation, while still helping to account for the impact of FV on AW's lives. Such measures cannot rule out all risks, and it remains incumbent on researchers to mitigate other possible threats to women's safety, including breaches of confidentiality when working in group settings. Researchers may also wish to review the resources to which Vaughan et al. (67) direct readers when engaging women having survived violence.

### 5.10. Integration of religion in clinical work

The consistency of findings around women's reliance on spirituality as a coping strategy may lead one to conclude that the integration of spiritual components during treatment is indicated. This is likely a reasonable assumption, though this may not be appealing to all women, and the suitability of religious components should thus be verified for all group members. Education may modulate religiosity and related coping strategies (106), so in addition to its other implications on client outcomes, educational attainment should be considered in all aspects of research design, including religious components. Moreover, respondents may feel socially obligated to overemphasize the importance of religion within their coping strategies, and women having survived life in a theocratic society may also have been injured by the rhetoric of religious figures, or they may hold dissenting political opinions (6, 36, 39, 206). Practitioners would therefore do well to gauge the degree of a client's religious attunement through means other than direct questioning.

### 5.11. Employment and educational programs

Although the sociopolitical environment in Afghanistan has long been perceived as entirely repressive to women, it's important to explore the creative ways in which women have chosen to empower themselves, given that many households have lost men's financial support to disaster, disability, unemployment, or substance dependence (36, 55, 80). Anecdotally, some women claim that enhancing their economic power through microfinance has also led to reduced beatings by their husbands (36). Since monetization of their activities has been seen to enhance financial stability or serve as a protective factor, individuals working with families could consider supporting women in seeking employment or exploring creative outlets that afford greater socialization within their communities. Granted, any program proposing work-based or educational interventions should consider benefits and risks, including an escalation of violence due to perceived imbalances, as Ackerson, et al. (207) have observed sometimes occurs when women from India receive more education relative to their husbands.

### 5.12. Reinforcing interventions through social impact

Any clinical approaches thus derived could also integrate elements of justice-focused or socially conscious models, whereby women can articulate how they would like societal wrongs to be addressed, since approaches of this variety can engender a sense of healing through constructive social critique (154, 157) Though a complex and growing field of inquiry, “Displaced persons should be recognised as critical stakeholders in transitional justice and reconciliation processes, and their arbitrary exclusion should be challenged” (208). Of note, as AW are frequently energized by the success and wellbeing of their children, framing these activities as being in the interest of future generations may also prove motivating (39).

### 5.13. Generalizability of resulting interventions

While it is possible that future research with AW could be applicable to resettled populations generally, the socioeconomic and environmental conditions that Afghans endorse may result a in a different set of thematic foci and formats as compared to groups from higher-income countries, where women may have greater socioeconomic mobility and a greater understanding of Western conceptualizations of MH. Though they would require tailoring, resulting interventions could be more applicable for women from lower-income countries having a greater number of environmental, GBV and conflict-related hazards. Other groups for whom such interventions may be applicable include those sharing ethnolinguistic commonalities, those from tribally organized societies, or those bearing religious similarities. They may also be more applicable to locations ranking high on the Political Terror Scale (209).

## 6. Limitations

Limitations of this article include: (1) constrained resources available for a more systematic identification of the gap; (2) only the English language literature is addressed, potentially overlooking local knowledge and language around MH which could be informative in developing tailored health systems; and (3) internal program implementation documents used in the establishment of systems such as the BPHS (118) are not considered due to lack of access to these materials.

## 7. Conclusion

The people of Afghanistan have endured significant violation of their self-determination from all manner of injustices, from foreign intervention in the form of sustained wars, the dependence on foreign support created by said wars, corruption within the Afghan government, and the presence of extremist ideological factions. All of these factors have had significant impacts on day-to-day living, amounting to what some scholars have called “interminable suffering” (210). Moreover, despite increased access to education and other social resources in the decades prior to the U.S. withdrawal, women continued to be further marginalized by pervasive abuse in the domestic and wider social spheres, compounding baseline trauma potentials within the environment.

Though findings on AW's health seeking patterns are sometimes inconsistent, ethnographic scholarship and other studies reveal a variety of reasons for which resettled Afghans are unable to fully utilize the health resources theoretically available to them. Critically, the present article finds that there is little information regarding clinical MH interventions for adult Afghans. AW are especially underrepresented in completed studies on interventions despite the substantial evidence surrounding their levels of distress and despite Afghanistan's overall interest to academia. Their health-seeking behaviors therefore require more research, especially since AW's agency and desires are more complex than some providers may presume them to be.

Though perhaps maintained by a prior lack of access to female research participants, this dearth poses challenges, as women's well-being may then be further compromised by health systems clamoring to help this cohort of high-profile arrivals, but which may lack the adequate knowledge base to do so. Many of the stressors ubiquitous to AW's experiences may persist upon resettlement, and untrained clinicians may interpret presentations as stemming primarily from the obvious war and resettlement trauma at the exclusion of other environmental factors, leading to inefficient targeting of concerns or to clients potentially feeling dismissed. Clients may also be subjected to inappropriate or potentially retraumatizing modalities, and “poor” service uptake could be incorrectly attributed to perceived cultural attributes or educational levels. Importantly, fragmented medical infrastructures can be discouraging and intrusive to persons not possessing the necessary experience with which to confidently navigate them (29). Therefore, AW's health-seeking and intervention preferences must be better understood in order to promote safe and equitable access to systems.

Given the 2021 humanitarian emergency, there is an urgent need to gather the understandings which had been painstakingly developed over the last two decades and validate them against a backdrop of radically changing political contexts. To help compensate for the lack of literature, this article synthesizes interdisciplinary material to provide greater context around women's frequently endorsed experiences, both in Afghanistan prior to the 2021 humanitarian crisis, as well as in resettled contexts. In so doing, it encourages the scholarly community to continue moving beyond studies on disorder prevalence and psychometrics, offering considerations which could be kept in mind when developing interventions for this population. Ideally, any such interventions would be derived through participatory research and would maintain a focus on integrated health, as well as family systems perspectives.

## Footnotes

^1^Afghanistan is highly pluralistic in terms of ethnolinguistic, religious, and tribal affiliations, and the country also has significant geographic variance (18–20). The term “Afghan” is used to refer to persons born in or habitually residing in Afghanistan and who also identify as such.

^2^Former president Hamid Karzai served from 2002 until 2014 (59).

^3^Providers may wish to consult the Gender-based Violence and Treatment Protocol for Healthcare in Providers in Afghanistan (72).

^4^As CBT and its variants can be easily adapted or integrated into other approaches, they are frequently used to address post-traumatic symptomatology, and there is often overlap between CBT-based methods and therapies targeting somatic aspects of client presentations (122); .however, a broad distinction is being made here between the structured, verbal approaches of CBT targeting psychological schema, as opposed to non-pharmacological, non-technological therapies expressly targeting dysregulation of the nervous system. An overview of some common trauma therapies can be found in State-of-the-Art Prevention and Treatment of PTSD (123).

^5^For individual work, see Knefel et al. (30).

^6^Omidian and Lawrence (149) have described a psychosocial program for aid workers in Afghanistan that integrated “focusing” techniques and spirituality. Though the perspectives afforded are insightful, the program is described as “non-clinical.” As such, the information from the publication is not presented in the format of a study providing access to detailed program methodology.

^7^Kananian et al. have registered a protocol for a CBT-based study involving Afghan men and women, among other groups (151).

^8^Limitations of this chapter include the fact that some aspects of the case, such as the client's low cognitive functioning, are not generalizable. The supernatural aspects of the client's interpretations, while representative of many Afghans, may also not be generalizable to all demographics.
